# Mapping the Literature on the Impact of Gastrointestinal Multiplexed Pathogen Panels on Clinical and Healthcare Utilization Outcomes: A Scoping Review

**DOI:** 10.1093/jalm/jfaf180

**Published:** 2025-12-16

**Authors:** Ahmed Babiker, N Esther Babady, A Brian Mochon, Amity L Roberts, Kileen L Shier, J Nicole Jackson, James Scott Parrott

**Affiliations:** Division of Infectious Diseases, Department of Internal Medicine, Rush University Medical Center, Chicago, IL, United States; Clinical Microbiology Service, Department of Pathology and Laboratory Medicine , Memorial Sloan Kettering Cancer Center, New York, NY, United States; Infectious Disease Service, Department of Medicine, Memorial Sloan Kettering Cancer Center, New York, NY, United States; Division of Pathology and Laboratory Medicine, Phoenix Children’s Hospital, Phoenix, AZ, United States; Department of Pathology and Laboratory Medicine, Hartford Hospital, Hartford CT, United States; Quest Diagnostics, Chantilly, VA, United States; American Society for Microbiology, Washington, DC, United States; Department of Interdisciplinary Studies, Rutgers School of Health Professions, Newark, NJ, United States

## Abstract

**Background:**

Gastrointestinal pathogen multiplex panels (GPPs) can test a single stool specimen for multiple pathogen targets in less than 5 h with some as little as 1 h. Although GPPs have demonstrated rapid and sensitive detection, their increased cost and unclear reimbursement structures have put into question their value and role in the cost-effective management of patients with gastrointestinal infections. We performed a scoping review to identify and systematically map the existing literature regarding the impact of GPPs on immediate, proximal, and distal clinical and healthcare utilization outcomes across healthcare settings.

**Methods:**

Databases were searched from inception until November 22, 2022. Full research articles in English were eligible for inclusion if they included a multiplexed (≥3 targets) nucleic acid amplification testing method and conventional microbiology comparator method performed on clinical samples.

**Results:**

A total of 6027 potential studies were identified. Following title and abstract screening and full article review, 175 studies were included. The most frequently studied GPPs were laboratory-developed tests (LDTs) (34.9%) and the Biofire^®^ FilmArray^®^ Gastrointestinal Panel (22.3%). The majority of these studies were conducted in the inpatient (37.1%), outpatient (28.0%), and emergency department (ED) (14.0%) settings. The most frequently reported outcomes included diagnostic accuracy (69.7%), organism detection (59%), time to diagnosis (12.6%), and antibiotic changes (8.6%).

**Conclusion:**

We identified a paucity of research reporting on proximal and distal outcomes associated with GPP use. Our review highlights the critical need for well-designed studies focusing on downstream clinical and healthcare utilization outcomes to guide meaningful and cost-effective incorporation of GPPs into diagnostic work flows.

IMPACT STATEMENT Timely and accurate detection of causative pathogens among patients with diarrheal illness can improve outcomes and interrupt disease transmission. By systematically mapping the existing literature, our scoping review summarizes the currently available evidence surrounding the clinical and healthcare utilization outcomes of gastrointestinal pathogen panels across healthcare settings. Our findings highlight the paucity of data and underscore the urgent need for well-designed studies to evaluate the cost-effectiveness and downstream benefits of GPPs, advancing diagnostic stewardship and guiding the meaningful and cost-effective incorporation of GPPs into diagnostic work flows.

## INTRODUCTION

Diarrheal diseases are a major cause of global mortality and morbidity (1). In 2021, 4.45 billion incident cases of enteric infections were reported (1). The WHO estimates diarrheal diseases are the third leading cause of death in children (1 to 59 months of age) globally (2). Acute gastroenteritis continues to exert a substantial burden upon healthcare systems across the United States with an estimated 47.8 million cases occurring annually, at an estimated healthcare cost exceeding $150 million (3, 4).

Timely and accurate detection and management of gastrointestinal (GI) pathogens can improve patient outcomes and interrupt disease transmission (5). The diagnosis of GI infections has traditionally relied on a combination of microbiological techniques, including microscopy, culture, antigen detection, and individual (singleplex) real-time PCR assays to detect bacteria, viruses, and parasites (6, 7). The development of commercial multiplex molecular diagnostic panels (target panels which test either up to 5 pathogen target panels or expanded panels which test ≥6 pathogen target panels) for the rapid detection of pathogens in positive blood culture bottles, respiratory specimens, stool, and cerebrospinal fluid has resulted in a paradigm shift in the work flow of infectious diseases laboratory diagnostics (8). These expanded syndromic panels (≥6 pathogen targets) allow healthcare providers to cast a broad net with rapid turnaround times and high sensitivity and specificity when compared to more traditional methods (8).

Gastrointestinal pathogen panels (GPPs) for the diagnosis of diarrheal illness may simultaneously test a single stool specimen for multiple bacterial, viral, and/or parasitic targets in less than 5 h, with some panels providing results in as little as 1 h (8). Although GPPs have demonstrated rapid and sensitive detection compared with conventional techniques, their increased cost and unclear payer reimbursement structures (in the United States) have put into question their specific role in the management of patients with diarrheal illness, especially in the emergency department (ED) and outpatient settings (9). Moreover, no guidance exists on how best to incorporate these panels into existing clinical microbiology work flows. The purpose of this scoping review was to identify and systematically map the existing literature evidence regarding the impact of GPPs on immediate, proximal, and distal health and healthcare utilization outcomes across healthcare settings.

## MATERIALS AND METHODS

The scoping review was conducted in accordance with the Joanna Briggs Institute Scoping Review Methodology group guidelines (10) and reported by adopting the preferred reporting items for systematic reviews and meta-analyses for scoping reviews (PRISMA-ScR) checklist (11). The purpose of this scoping review was to identify areas of research where there would likely be sufficient research to carry out a subsequent series of systematic reviews. Hence, no protocol was registered for this scoping review.

### Eligibility Criteria

To be included in the scoping review, papers needed to meet the following eligibility criteria:

1. Include a multiplexed (≥3 GI pathogens excluding *Clostridioides difficile*) nucleic acid amplification test (NAAT) method and conventional microbiology assay comparator (microscopy, antigen testing, bacterial or viral culture, and/or singleplex PCR) method.
2. Stool testing was performed on clinical (patient-derived) samples, rather than contrived samples (e.g., synthetic, spiked, or laboratory-manipulated specimens).
3. Full primary research article using a comparative design (e.g., no conference abstracts, commentaries, reviews, or case studies).
4. Full article available in English.

Studies that did not meet these criteria were screened out and data were not included.

### Search Criteria and Information Sources

To identify potentially relevant documents, MEDLINE, EMBASE, and Cochrane were searched from inception to November 22, 2022. The search strategy was constructed by a medical librarian and further refined through team discussion. The full syntax for search strategy can be found in the supplement (Supplemental data).

### Screening and Data Charting Process

Citations for all articles were imported into COVIDENCE for initial title and abstract screening. Eligible citations were then imported into the US Agency for Healthcare Research and Quality Systematic Review Data Repository Plus (SRDR+) tool for full article review. Studies were simultaneously screened (using a series of screening questions at the beginning of the extraction template) and, if study inclusion criteria were met, data were blind extracted by 2 or more members of the team. Both screening and data extraction responses were adjudicated by a third team member in the case of disagreement.

### Data Items and Presentation

Data on the following study characteristics were extracted: (*a*) population studied (age group, practice setting, health condition, and diarrheal syndrome), (*b*) GPP method/assay utilized, (*c*) comparator diagnostic tests utilized, (*d*) type of pathogens (bacterial, viral, or parasite) both tested for and detected, and (*e*) outcomes. Outcomes were categorized into immediate outcomes (diagnostic accuracy, time to diagnosis, detection/yield/prevalence, or other), proximal health outcomes (time to therapy, length of stay, mortality, change in antibiotic, isolation/infection control actions, infection resolution, hospital admission and readmission, or other changes in therapy), and distal outcomes (cost or cost/benefit, reimbursement, outbreak management, or other).

### Analysis

Results are presented as heatmaps of the number of cross-classified studies and Sankey charts. Figure generation and descriptive analyses were carried out in R version 4.3.3.

## RESULTS

### Selection of Evidence

**Fig. 1.**
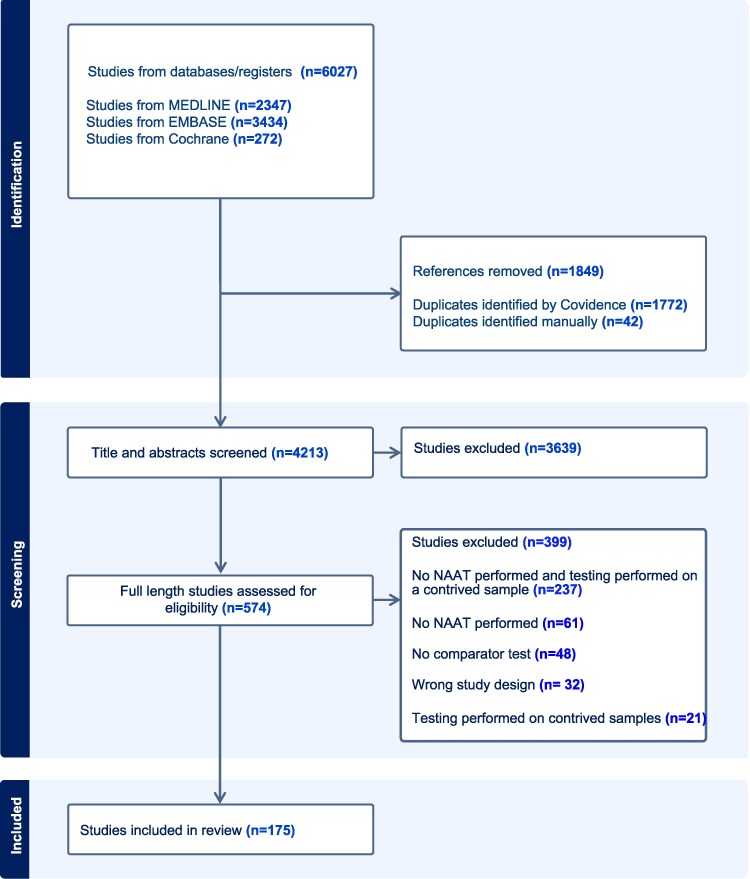
PRISMA study flowsheet. Abbreviations: NAAT, nucleic acid amplification test; PRISMA, Preferred reporting items for systematic reviews and meta-analyses.

A total 6027 studies were identified from searches of electronic databases. After duplicates were removed (*n* = 1814), a total of 4213 citations remained. Based on the title and the abstract, 3639 studies were excluded, with 574 full text articles to be retrieved and assessed for eligibility. Of these, 399 were excluded for the following reasons: no NAAT performed and testing performed on a contrived sample (*n* = 237), no NAAT performed (*n* = 61), no comparator test (*n* = 48), wrong study design (*n* = 32), and testing performed on contrived sample (*n* = 21). The remaining 175 studies were considered eligible for this review (Fig. 1).

### Study Characteristics

Studies were grouped by GPP utilized, healthcare setting, and outcomes (immediate, proximal, and distal). The characteristics of the included studies are summarized in Table 1.

**Table 1.** Characteristics of eligible studies (*n* = 175).

Variables	*n* (%)
Multiplex panels	
LDT	61 (34.9)
Other commercial multiplex	43 (24.6)
FilmArray Gastrointestinal Panel	39 (22.3)
XTAG GPP	33 (18.9)
BDMAX Bacteria	14 (8.0)
BDMAX Parasite	3 (1.7)
BDMAX Viral	2 (1.1)
Verigene	2 (1.1)
Settings	
Inpatient	65 (37.1)
Outpatient	49 (28.0)
Emergency department	24 (14.0)
Travelers or refugees	11 (6.3)
Community acquired	11 (6.3)
Long-term care	3 (1.7)
Comparator panels	
Bacterial culture	107 (61.1)
Alternative NAAT	91 (52.0)
Antigen/EIA	77 (44.0)
Parasite microscopy	71 (40.6)
Other comparator	21 (12.0)
Viral culture	2 (1.1)
Immediate outcomes	
Diagnostic accuracy	122 (69.7)
Detection	95 (54.2)
Time to diagnosis	22 (12.6)
Other immediate outcomes	9 (5.1)
Proximal outcomes	
Antibiotic change	15 (8.6)
Other proximal outcome	10 (5.7)
Hospital admission	8 (4.6)
Infection resolution	7 (4.0)
Infection control intervention	5 (2.9)
Time to appropriate treatment	4 (2.3)
Length of stay	4 (2.3)
Mortality	1 (0.6)
Distal outcomes	
Cost	10 (5.5)
Other distal outcome	3 (1.7)

Abbreviations: EIA, enzyme immunoassay; LDT, laboratory-developed test; GPP, gastrointestinal panel; NAAT, nucleic acid amplification test.

**Fig. 2.**
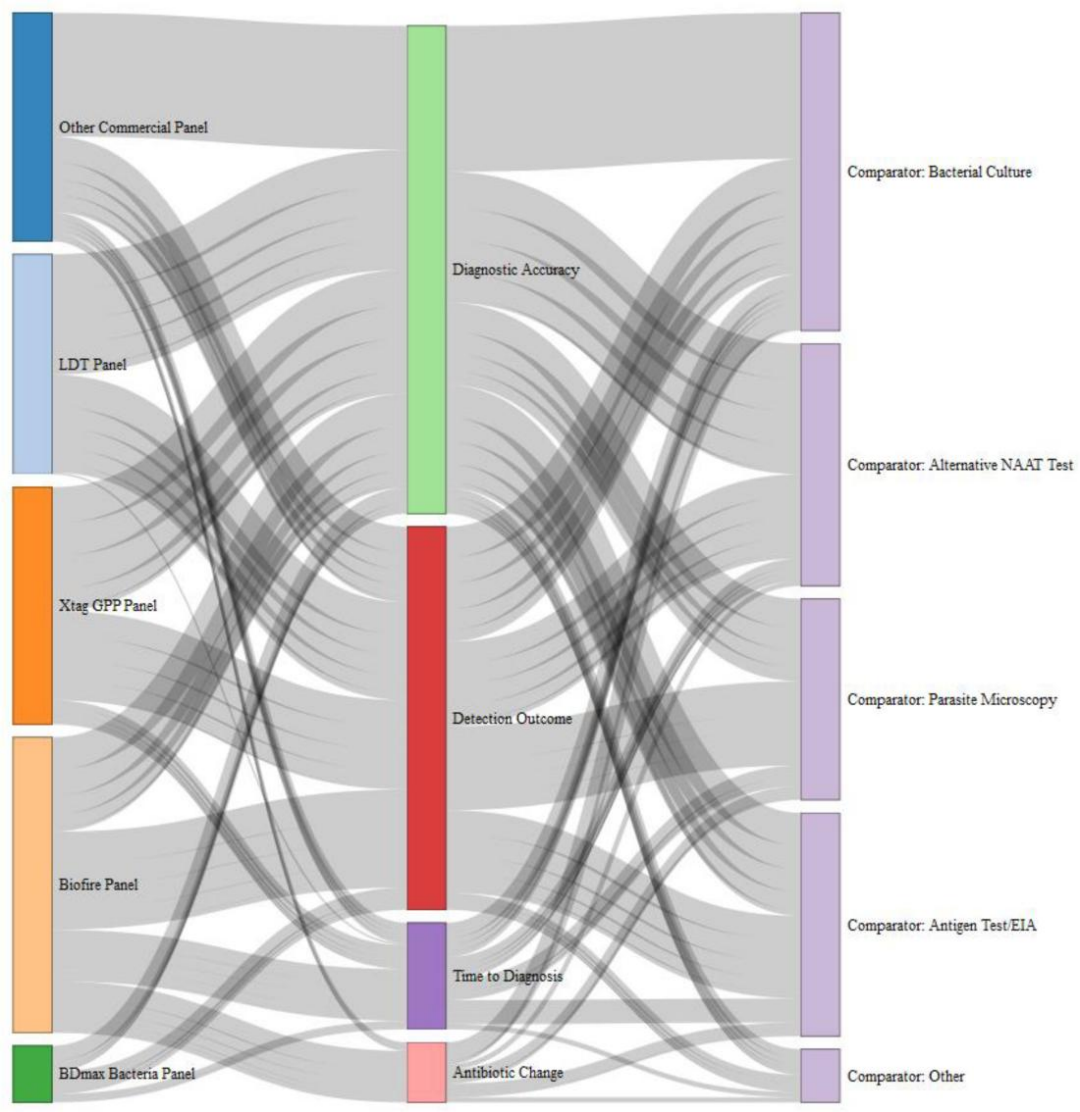
**Sankey chart of studies reporting on most commonly reported outcomes by gastrointestinal pathogen panel and comparator. Sankey charts visualize flow between categories using proportional links: the width of each line represents the relative frequency of studies transitioning between categories (e.g., gastrointestinal multiplex pathogen panel to outcome domain to comparator category). Abbreviations: EIA, enzyme immunoassay; NAAT, nucleic acid amplification test; GPP, gastrointestinal pathogen panel**.

The most frequently studied GPPs were laboratory-developed tests (LDTs) (*n* = 61 studies, 34.9%). The most frequently studied commercially available GPPs included the BIOFIRE FILMARRAY GI Panel (BioFire Diagnostics) (*n* = 39 studies, 22.3%), the xTAG^®^ GPP (Luminex), (*n* = 33 studies, 18.9%), and the BD MAX™ Enteric Bacterial Panel (Becton Dickinson) (*n* = 14 studies, 8.0%). Forty-three studies (24.6%) evaluated “other” commercial GPPs (Table 1, Fig. 2) that were available outside of the United States. Comparator modalities included bacterial culture (*n* = 107 studies, 61.1%), alternative NAAT (*n* = 91 studies, 52%), antigen/enzyme immunoassay (EIA) testing (*n*= 77 studies, 44%), microscopy for parasite detection (*n* = 71 studies. 40.6%), “other” miscellaneous test (*n* = 21 studies, 12%) and viral culture (*n* = 2 studies, 1.1%); see Table 1. Studies could include more than one comparator test modality (Fig. 2).

### Immediate Outcomes

**Fig. 3.**
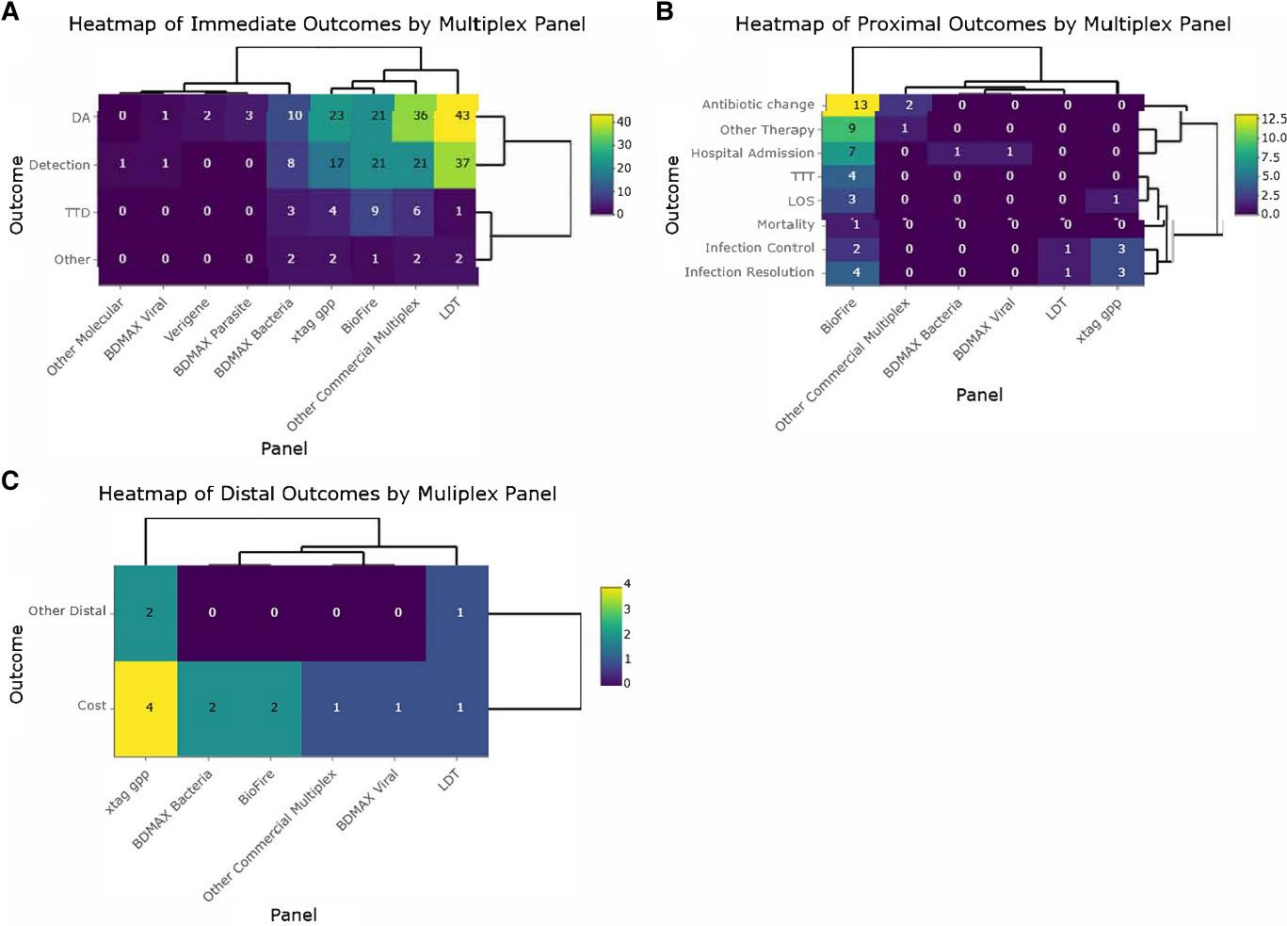
**Heatmap of immediate outcomes (A), proximal outcomes (B), and distal outcomes (C) by gastrointestinal pathogen panel. Abbreviations: DA, diagnostic accuracy; LDT, laboratory-developed test; LOS, length of stay; TTT, time to appropriate therapy; TTD, time to diagnosis**.

One hundred sixty-seven studies (of *n* = 175, 95.4%) met eligibility criteria and reported immediate outcomes of interest. Immediate outcomes included diagnostic accuracy (*n* = 122 studies, 69.7%), detection (*n* = 131 studies, 59%), time to diagnosis (*n* = 22 studies, 12.6%) and “other” immediate outcomes (*n* = 9 studies, 5.1%) (Fig. 3A). Just over a third of the studies reporting immediate outcomes reported multiple (*n* = 66 studies, 39.5%).

Sufficient research to perform further subsequent analysis for 2 immediate outcomes was identified; diagnostic accuracy and detection rate (Table 1). There may be a sufficient number of studies to differentiate performance by GPP for a limited number of panels and immediate outcomes. These include the BIOFIRE FILMARRAY GI Panel (diagnostic accuracy: *n* = 21 studies, detection: *n* = 21 studies, time to diagnosis: *n* = 9 studies), the xTAG GPP (diagnostic accuracy: *n* = 23 studies, detection: *n* = 17 studies), and, to a lesser degree, the BD MAX Enteric Bacterial Panel (diagnostic accuracy: *n* = 10 studies, detection: *n* = 8 studies) (Fig. 3A).

### Proximal Outcomes

Twenty-two studies met eligibility criteria and reported proximal outcomes of interest. Proximal outcomes included antibiotic change (*n* = 15 studies, 8.6%), hospital admission (*n* = 8 studies, 4.6%), infection resolution (*n* = 7 studies, 4.0%), infection control intervention (*n* = 5, 2.9%), time to appropriate treatment (*n* = 4, 2.3%), length of stay (*n* = 4, 2.3%), mortality (*n* = 1, 0.6%) and “other” proximal outcomes. (*n* = 10, 5.7%). Some studies reported multiple proximal outcomes (*n* = 18 studies, 81% of studies reporting any proximal outcome). No studies reported on hospital readmission or morbidity outcomes. (Table 1, Fig. 3C).

Amongst studies that reported proximal outcomes, the FilmArray GI panel was the most commonly evaluated GPP (Fig. 3B). Further analysis for the outcome antibiotic change could be performed for the FilmArray GI panel (*n* = 13 studies) (Fig. 3B).

### Distal Outcomes

Twelve studies met eligibility criteria and reported distal outcomes of interest. These included healthcare cost (*n* = 10 studies, 5.5%) and “other” distal outcomes. (*n* = 3, 1.7%) (Table 1, Fig. 3C). Only one study reported more than one distal outcome (8% of studies reporting any distal outcome).

No studies reporting reimbursement or other distal outcomes were identified. Given the low number of studies reporting on distal outcomes, no meaningful evaluation of distal outcomes by panel or setting is likely.

### Study Settings

Eligible studies that reported on setting included studies conducted in the inpatient setting (*n* = 65 studies, 37.1%), outpatient setting (*n* = 49 studies, 28.0%), ED setting (*n* = 24 studies, 14.0%), and studies conducted among travelers and/or refugees (*n* = 11 studies, 6.3%), patients with community acquired diarrhea (*n* = 11 studies, 6.3%), and residents of long-term care facilities (*n* = 3 studies, 1.7%) (Table 1). Some studies did not clearly report on setting (*n* = 78, 44.5%), while some studies reported on outcomes in more than one setting (*n* = 43, 55.4%).

Three settings (inpatient, outpatient, and ED) are likely to have a sufficient number of studies to make meaningful comparisons within a setting for a limited number of outcomes. These include diagnostic accuracy and detection in the inpatient setting (diagnostics accuracy: *n* = 41 studies, detection *n* = 37 studies), outpatient setting (diagnostics accuracy: *n* = 30 studies, detection *n* = 28 studies), and the ED setting (diagnostic accuracy *n* = 16 studies, detection: *n* = 13 studies). A limited evaluation may be possible for the time to diagnosis (TTD) outcome in the inpatient setting (*n* = 13 studies) and outpatient setting (*n* = 7 studies). Evaluation of the diagnostic accuracy of the FilmArray GI panel and to a lesser extent the xTAG GPP within the inpatient, outpatient, and ED settings may be possible.

Amongst studies that reported on proximal outcomes, further analysis by setting could be possible for the outcome antibiotic change in the inpatient (*n* = 11 studies) and outpatient (*n* = 8 settings. Analysis by setting is likely not possible for distal outcomes.

## DISCUSSION

GPPs allow clinicians to cast a broad net and achieve a timely diagnosis (12). Sample-to-answer GPPs have become increasingly utilized due to several advantages over conventional methods including, but not limited to, decreased technician hands-on time, broad agnostic coverage negating the need for upfront selection of appropriate tests, enhanced coinfection detection, increased sensitivity over some conventional microbiological approaches, and reduced turnaround time (12). The purpose of this scoping review was to identify areas where there would be sufficient research to allow systematic reviews to evaluate the clinical impact of GPPs. We identified 175 primary studies that reported on clinical and healthcare utilization outcomes of interest published from databases inception to 2022. The vast majority of these focused on the immediate performance outcomes such as diagnostic accuracy and detection rate. Research on meaningful proximal and distal clinical and healthcare utilization outcomes was limited.

Despite the advantages that GPPs offer over conventional microbiology work flows, questions exist regarding the impact of GPPs on important patient and healthcare utilization outcomes. The majority of studies identified, which compared GPPs to conventional microbiology assay comparators, were centered around diagnostic accuracy and pathogen detection rate. Molecular testing modalities typically offer a higher degree of sensitivity than conventional tests (13). However, molecular assays detect nucleic acid and cannot distinguish between viable and nonviable organisms, which may be detected for long periods of time following resolution of the infection or may represent colonization (14). Whether the use of more sensitive assays, which leads to increased and earlier detection of organisms, leads to improved downstream outcomes is yet to be robustly demonstrated with GPPs (15–17). With similar multiplex panels, clinical outcomes are generally impacted when coupled with stewardship interventions and guidance on results interpretation (18). Studies on conventional clinical and healthcare utilization outcomes such as mortality (one study), morbidity (no studies), hospital admission (eight studies), hospital readmission (no studies), time to appropriate treatment (four studies), or length of stay (four studies) were limited or absent.

The implementation and use of GPPs leads to increased upfront costs when compared to routine diagnostics applied in the evaluation of a patient with diarrhea (19). However, the use of a single multiplex assay may be more cost-effective if performed in lieu of multiple conventional tests (19, 20). Moreover, there is a potential for important healthcare utilization cost savings such as decreased antibiotic exposures and decreased length of isolation, length of stay, and admissions/readmissions (19). While we identified a limited number of studies that reported on costs (10 studies), cost-effectiveness analysis studies are notoriously heterogeneous, and so it is questionable if these studies could be confidently aggregated and analyzed or extrapolated to other settings. Notably, reimbursement was rarely reported across studies, despite its relevance as a potential barrier to GPP implementation. Further studies that demonstrate the overall cost-effectiveness offered to the healthcare system are needed to justify the added healthcare operational expenditure associated with GPPs.

We found the majority of studies identified utilized LDTs. LDTs, long a component of standard laboratory operations, have historically been developed and utilized with minimal regulatory oversight by the FDA due to perceived low risk and CLIA regulatory oversight (21). In May 2024, the FDA issued a final rule to expand its regulatory authority over LDTs, intending to treat them as medical devices subject to FDA premarket review. However, in March 2025, a federal district court vacated the rule, concluding that the FDA lacked the statutory authority to regulate LDTs in this manner. As of September 2025, the FDA has formally rescinded the rule, restoring the previous regulatory landscape. These developments have created renewed uncertainty regarding the future regulation of LDTs and raised questions about test accessibility, compliance burdens, and downstream research (22). Moreover, the LDT panels studied represent a heterogenous group, which differ in terms of target organisms. Studies utilizing FDA approved panels were less frequent and dominated by 2 panels (the Filmarray GI and xTAG panels), likely a reflection of the recency of these panels, limiting further study of outcomes extrapolation.

The burden and risk of diarrheal illness can vary widely across different patient populations (23). The majority of studies identified in our review were conducted in the inpatient, outpatient, and ED settings, representative of the range of severity of diarrheal illness (5). This will hopefully allow for further analysis (depending on the selected outcome) by setting. This is of importance as etiology and diagnostic approach can differ based upon the practice setting. Our review highlights the need for further studies in clearly defined settings with a high burden of diarrheal illness and potential for a wider range of infectious agents, such as in low- and middle-income settings, and among distinct patient populations such as traveler and refugee populations and immunosuppressed patients to guide more specific and targeted implementation of GPPs.

Our scoping review has some limitations. We only included English peer-reviewed research and omitted the gray literature, limiting the number of included studies. In addition, we limited our review to studies that included a direct comparator method to evaluate the potential impact that GPPs offer when compared to existing diagnostics processes. While the widespread adoption of GPPs makes it increasingly challenging to conduct studies using a traditional comparator method, investigators can potentially utilize pre-post-intervention quasi-experimental study designs, which can leverage historical data from the pre-GPP period and compare it to outcomes after GPP implementation (24, 25).

The purpose of this scoping review was to identify areas of research where there would likely be sufficient research to carry out a subsequent series of systematic reviews. Based on our findings, we anticipate that focused systematic reviews may be feasible for certain outcome domains, such as diagnostic accuracy, detection rate, time to diagnosis, and antibiotic change, particularly for more commonly used FDA-cleared panels. We intend to pursue these endpoints for targeted reviews, to better quantify the impact of GPPs on clinical and healthcare utilization outcomes to inform evidence-based utilization strategies.

In conclusion, our scoping review identified a paucity of research reporting on meaningful downstream proximal and distal health outcomes associated with the use of GPPs. Generation of such data with well-designed studies is of paramount importance to guide the rationale and mindful integration of GPPs into clinical care work flows.

## Supplemental Material

Supplemental material is available at *The Journal of Applied Laboratory Medicine* online.

## Supplementary Material

jfaf180_Supplementary_Data

## Notes

**Nonstandard Abbreviations:** GPP, gastrointestinal pathogen panel; LDT, laboratory-developed test; ED, emergency department; WHO, World Health Organization; GI, gastrointestinal; PRISMA-ScR, preferred reporting items for systematic reviews and meta-analyses for scoping reviews; NAAT, nucleic acid amplification test; SDRD+, Systematic Review Data Repository Plus; EIA, enzyme immunoassay; TTD, time to diagnosis.

**Author Contributions:**  *The corresponding author takes full responsibility that all authors on this publication have met the following required criteria of eligibility for authorship: (a) significant contributions to the conception and design, acquisition of data, or analysis and interpretation of data; (b) drafting or revising the article for intellectual content; (c) final approval of the published article; and (d) agreement to be accountable for all aspects of the article thus ensuring that questions related to the accuracy or integrity of any part of the article are appropriately investigated and resolved. Nobody who qualifies for authorship has been omitted from the list*.

**Authors’ Disclosures or Potential Conflicts of Interest:**  *Upon manuscript submission, all authors completed the author disclosure form.*

**Research Funding:** A. Babiker was supported in part by an Antibacterial Resistance Leadership Group Early Faculty Seedling Award [National Institute of Allergy and Infectious Diseases UM1AI104681]. N.E. Babady was supported in part through the National Cancer Institute Cancer Center Support Grant P30 CA008748.

**Disclosures:** A. Babiker reports serving on a clinical board advisory for Beckman Coulter. N.E. Babady reports serving on a scientific advisory board for Roche/GenMark and Bio-Rad. K.L. Shier is an employee of Quest Diagnostics.

**Role of Sponsor:** The funding organizations played no role in the design of study, choice of enrolled patients, review and interpretation of data, preparation of manuscript, or final approval of manuscript.
