# Supplementary material for: Mapping the Literature on the Impact of Gastrointestinal Multiplexed Pathogen Panels on Clinical and Healthcare Utilization Outcomes: A Scoping Review
Source: J Appl Lab Med. 2025 Dec 16;11(2):269–80. doi: 10.1093/jalm/jfaf180 (PMC12776536; doi:10.1093/jalm/jfaf180)
Supplement: jfaf180_Supplementary_Data [file jfaf180_supplementary_data.docx]

**Search Strategy**

MEDLINE, PubMed

("Infections"[Mesh] OR infection*[tiab] OR infectious[tiab] OR pathogen*[tiab] OR organism*[tiab] OR microb*[tiab] OR bacterial[tiab] OR viral[tiab] OR parasitic[tiab] OR parasitosis[tiab] OR fungal[tiab] OR mycosis[tiab])

AND

("Multiplex Polymerase Chain Reaction"[Mesh] OR "multiplex polymerase chain reaction"[tiab] OR "multiplex PCR"[tiab] OR "multiplex real-time polymerase chain reaction"[tiab] OR "multiplex RT-PCR"[tiab] OR '"triplex polymerase chain reaction"[tiab] OR "triplex PCR"[tiab] OR "triplex real-time polymerase chain reaction"[tiab] OR "triplex real-time PCR"[tiab] OR "triplex RT-PCR"[tiab] OR ((multiplex[tiab] OR triplex[tiab]) AND (PCR[tiab] OR RT-PCR[tiab] OR qRT-PCR[tiab] OR "polymerase chain reaction"[tiab])) OR "gastrointestinal pathogen panel*"[tiab] OR "gastrointestinal pathogen test*"[tiab] OR "enteric pathogen panel*"[tiab] OR "enteric pathogen test*"[tiab] OR "enteric bacterial panel*"[tiab] OR "enteric viral panel*"[tiab])

AND

("Gastroenteritis"[Mesh] OR "Diarrhea"[Mesh] OR "Feces"[Mesh] OR gastroenteritis[tiab] OR diarrhea*[tiab] OR diarrhoea*[tiab] OR enteric*[tiab] OR enteritis[tiab] OR colitis[tiab] OR gastrointestin*[tiab] OR stool*[tiab] OR feces[tiab] OR faeces[tiab] OR fecal[tiab] OR faecal[tiab])

Results: 2,341

Date Ran: 12/1/2022

Date Uploaded: 12/15/2022

Embase, Elsevier

('infection'/exp OR infection*:ti,ab OR infectious:ti,ab OR 'infectious agent'/exp OR pathogen*:ti,ab OR 'microorganism'/exp OR microbe*:ti,ab OR 'bacterial infection'/exp OR bacterial:ti,ab OR 'virus infection'/exp OR viral:ti,ab OR 'mycosis'/exp OR fungal:ti,ab OR mycosis:ti,ab OR 'parasitosis'/exp OR parasitic:ti,ab OR parasitosis:ti,ab)

AND

('multiplex polymerase chain reaction'/exp OR 'multiplex polymerase chain reaction':ti,ab OR 'multiplex PCR':ti,ab OR 'multiplex real-time polymerase chain reaction':ti,ab OR 'multiplex real-time PCR':ti,ab OR 'multiplex RT-PCR':ti,ab OR 'triplex polymerase chain reaction':ti,ab OR 'triplex PCR':ti,ab OR 'triplex real-time polymerase chain reaction':ti,ab OR 'triplex real-time PCR':ti,ab OR 'triplex RT-PCR':ti,ab OR ((multiplex:ti,ab OR triplex:ti,ab) AND (PCR:ti,ab OR RT-PCR:ti,ab OR qRT-PCR:ti,ab OR 'polymerase chain reaction':ti,ab)) OR 'gastrointestinal pathogen panel*':ti,ab OR 'gastrointestinal pathogen test*':ti,ab OR 'enteric athogen panel*':ti,ab OR 'enteric pathogen test*':ti,ab OR 'enteric bacterial panel*':ti,ab OR 'enteric viral panel*':ti,ab)

AND

('gastroenteritis'/exp OR gastroenteritis:ti,ab OR gastrointestin*:ti,ab OR 'diarrhea'/exp OR diarrhea*:ti,ab OR diarrhoea*:ti,ab OR 'feces'/exp OR feces:ti,ab OR faeces:ti,ab OR fecal:ti,ab OR faecal:ti,ab OR enteric*:ti,ab OR OR enteritis:ti,ab OR colitis:ti,ab OR stool*:ti,ab)

Results: 3,372

Date Ran: 12/1/2022

Date Uploaded: 12/15/2022

Cochrane Library, Wiley

((infection OR infectious OR pathogen* OR microorganism* OR microbe* OR bacteria infection* OR virus infection* OR bacterial OR viral OR fungal OR mycosis OR parasitic OR parasitosis)):ti,ab,kw

AND

((multiplex polymerase chain reaction OR multiplex PCR OR multiplex real-time polymerase chain reaction OR multiplex real-time PCR OR multiplex RT-PCR OR triplex polymerase chain reaction OR triplex PCR OR triplex real-time polymerase chain reaction OR triplex real-time PCR OR triplex RT-PCR OR ((multiplex OR triplex) AND (PCR OR RT-PCR OR qRT-PCR OR polymerase chain reaction)) OR gastrointestinal pathogen panel* OR gastrointestinal pathogen test* OR enteric pathogen test* OR enteric bacterial panel* OR enteric viral panel*)):ti,ab,kw

AND

((gastroenteritis OR gastrointestin* OR diarrhea* OR diarrhoea* OR feces OR faeces OR fecal OR faecal OR enteric* OR enteritis OR colitis OR stool)):ti,ab,kw

Results: 273 (5 reviews, 268 trials)

Date Ran: 12/1/2022

Date Uploaded: 12/15/2022
